# Design and Conceptual Proposal of an Intelligent Clinical Decision Support System for the Diagnosis of Suspicious Obstructive Sleep Apnea Patients from Health Profile

**DOI:** 10.3390/ijerph20043627

**Published:** 2023-02-18

**Authors:** Manuel Casal-Guisande, María Torres-Durán, Mar Mosteiro-Añón, Jorge Cerqueiro-Pequeño, José-Benito Bouza-Rodríguez, Alberto Fernández-Villar, Alberto Comesaña-Campos

**Affiliations:** 1 Department of Design in Engineering, University of Vigo, 36208 Vigo, Spain; 2 Design, Expert Systems and Artificial Intelligent Solutions Group (DESAINS), Galicia Sur Health Research Institute (IIS Galicia Sur), SERGAS-UVIGO, 36213 Vigo, Spain; 3 Pulmonary Department, Hospital Álvaro Cunqueiro, 36213 Vigo, Spain; 4 NeumoVigo I+i Research Group, Galicia Sur Health Research Institute (IIS Galicia Sur), SERGAS-UVIGO, 36213 Vigo, Spain

**Keywords:** obstructive sleep apnea, design, intelligent system, Machine Learning, neuro-fuzzy inference system, heuristics, clinical decision support system, medical algorithm, medical decision-making

## Abstract

Obstructive Sleep Apnea (OSA) is a chronic sleep-related pathology characterized by recurrent episodes of total or partial obstruction of the upper airways during sleep. It entails a high impact on the health and quality of life of patients, affecting more than one thousand million people worldwide, which has resulted in an important public health concern in recent years. The usual diagnosis involves performing a sleep test, cardiorespiratory polygraphy, or polysomnography, which allows characterizing the pathology and assessing its severity. However, this procedure cannot be used on a massive scale in general screening studies of the population because of its execution and implementation costs; therefore, causing an increase in waiting lists which would negatively affect the health of the affected patients. Additionally, the symptoms shown by these patients are often unspecific, as well as appealing to the general population (excessive somnolence, snoring, etc.), causing many potential cases to be referred for a sleep study when in reality are not suffering from OSA. This paper proposes a novel intelligent clinical decision support system to be applied to the diagnosis of OSA that can be used in early outpatient stages, quickly, easily, and safely, when a suspicious OSA patient attends the consultation. Starting from information related to the patient’s health profile (anthropometric data, habits, comorbidities, or medications taken), the system is capable of determining different alert levels of suffering from sleep apnea associated with different apnea-hypopnea index (AHI) levels to be studied. To that end, a series of automatic learning algorithms are deployed that, working concurrently, together with a corrective approach based on the use of an Adaptive Neuro-Based Fuzzy Inference System (ANFIS) and a specific heuristic algorithm, allow the calculation of a series of labels associated with the different levels of AHI previously indicated. For the initial software implementation, a data set with 4600 patients from the Álvaro Cunqueiro Hospital in Vigo was used. The results obtained after performing the proof tests determined ROC curves with AUC values in the range 0.8–0.9, and Matthews correlation coefficient values close to 0.6, with high success rates. This points to its potential use as a support tool for the diagnostic process, not only from the point of view of improving the quality of the services provided, but also from the best use of hospital resources and the consequent savings in terms of costs and time.

## 1. Introduction

Obstructive sleep apnea (OSA) is a chronic pathology affecting about one thousand million people worldwide [1]. It is characterized by recurrent episodes of total or partial collapse of the upper airways during sleep which impairs sleep quality, producing fatigue symptoms and daytime sleepiness. In spite of its diagnostic difficulty, it is a treatable pathology using specific therapeutic approaches such as positive airway therapies, which alleviate the symptoms of the disease. However, if not treated, it will become detrimental to the patient’s health, causing the development of arterial hypertension, increased risk of heart and cerebrovascular events, as well as cognitive and metabolic alterations.

The golden standard technique for the diagnosis of OSA is in-lab polysomnography [2,3,4,5,6,7], which consists in a series of physiological measurements during sleep that allow characterizing the presence of the pathology. Nevertheless, there are other cheaper and simpler alternatives for the diagnosis of this pathology, such as cardiorespiratory polygraphy; however, it does not collect information on neurophysiological variables [8,9,10].

After these studies, the next main metric used for assessing OSA severity is often the apnea-hypopnea index (AHI), which relates to the number of apnea and hypopnea events recorded in an overnight sleep study divided by the hours of total sleep [6,11]. In this case, apnea would be defined as the complete interruption of respiratory function for 10 or more seconds, while hypopnea would refer to a decrease of respiratory flow greater than 30% for 10 s or more, accompanied by micro-awakening or desaturation below 4% [12,13].

Making the problem even worse, the symptoms presented by this type of patient are not very specific or unusual in the general population (excessive sleepiness, snoring, etc.), so it is common for many patients who are referred for a sleep test to receive a non-OSA result. This highlights and points to the need for more and better screening processes, and to reduce the number of patients referred to units specialized in respiratory sleep pathologies, allowing the prioritization of those who really need it, thus resulting, besides the relevant diagnostic and therapeutic advantages, in a significant reduction in associated costs.

In this sense, in recent decades, different types of tools and approaches have been developed and proposed for the screening of patient candidates for an OSA case, both based on the completion of questionnaires supported by medical criteria, and on approaches inspired by artificial intelligence techniques that aim to help in the early detection of those patients having the highest risk of suffering from the pathology. Among the different types of questionnaires, we could highlight the Berlin questionnaire, the STOP-Bang questionnaire, and the STOP questionnaire which have been widely used to detect the pathology [14], presenting high sensitivity and low specificity [15]. In relation to the approaches and techniques supported by artificial intelligence and based on patient clinical data (i.e., demographic information, comorbidities, or their symptoms), their aim is to identify and characterize those patients who could suffer from an OSA case, which would simplify the diagnostic process, making it more affordable and convenient. For example, the work by Corrado Mencar et al. [16] evaluated the effectiveness and applicability of Machine Learning approaches to determine the degree of severity of OSA suffered by a patient. To do this, they started from a data set associated with 313 patients treated in two Italian sleep units. This set included data from the patients’ history and others derived from the patients’ answers to some specific questionnaires. On the one hand, they applied classification approaches which gave a maximum precision rate applying k-fold cross validation of 44.7%, while on the other, regression approaches were used to determine the apnea-hypopnea index (AHI), obtaining a minimum root mean square error value of 22.17. In the work by Berk Ustun et al. [17], using a data set containing variables obtained from the history and symptoms of 1922 patients who underwent sleep tests in the United States of America, the use of different Machine Learning approaches was analyzed, such as logistic regression variants, decision trees, support vector machines or Supersparse Linear Integer Models (SLIM). The authors point out that better results are obtained when only the information from the patient’s medical history (demographic data and comorbidities) is used, than when only the information related to the symptoms associated with OSA is used. They mention that the performance of the different algorithms was similar, with values for sensitivity [18] of 64.2% and for specificity [18] of 77% in the case of SLIM. Similarly, in the work by Jayroop Ramesh et al. [19], based on a data set with 1479 patients from the Wisconsin Sleep Cohort data set that includes information about demographics, anthropometry, blood tests, derived clinical markers, general health questionnaires, self-reported history and polysomnography-derived parameters, the performance of different Machine Learning approaches was analyzed. In that work, the best results were shown for support vector machines, with values for sensitivity of 88.76% and for specificity of 40.74%. In the work by Wen-Chi Huang et al. [20], starting from a data set of 6875 Chinese patients who underwent sleep testing for suspected OSA and that included information on demographics, anthropometrics, comorbidities, self-reported habitual sleep patterns, and OSA symptoms, the performance of a series of vector support machines was analyzed for the classification of patients with AHI levels greater than 5, 15 and 30. After their training and analysis, the obtention of area under the curve values [21] of 0.82, 0.80 and 0.78, sensitivity values of 74.14%, 75.18%, and 70.26%, and specificity values of 74.71%, 68.73%, and 70.30%, respectively, were observed for the previously mentioned classifiers.

In addition to what has already been specifically mentioned for the diagnosis of OSA, numerous approaches are used in medical diagnosis that use and combine different Machine and Deep Learning algorithms, among which we could highlight: convolutional neural networks (CNN) [22,23,24,25,26,27], recurrent neural networks (RNN) [28,29,30,31,32], genetic algorithms (GA) [33,34,35], clustering approaches [36,37,38,39] or neuro-symbolic approaches, such as the adaptive neuro fuzzy inference system (ANFIS) [40,41,42,43].

Likewise, it is necessary to point out that the usual and most common approach to compare and determine the effectiveness of this type of predictive systems is to understand them as binary classifiers [44,45]. A binary classifier intends to determine the relationships between both the properly classified cases—those that the classifier has succeeded—and the erroneously classified—those that the classifier has failed— within a data set labeled with two possible classes. From the information related to the successes and failures of the classifier, four metrics are generally established from which it is possible to build the confusion matrix—true positives (TP), true negatives (TN), false positives (FP) and false negative (FN)—that are used to calculate the sensitivity, specificity, receiver operating characteristic curve (ROC), as well as the area under that curve (AUC), the accuracy or F1-score of the binary classifier. In the case of having an unbalanced data set, which is common in medical settings, it is usually advisable to replace the metrics related to the accuracy of the model by the Matthews correlation coefficient (MCC), a particularization of the Phi coefficient [46,47,48], which allows measuring the performance of the classifier in a more reliable way, only providing a high score if satisfactory results were obtained in the four metrics that characterize the confusion matrix [47]. In this sense, the Matthews correlation coefficient value lies in the interval [−1 1], with −1 and 1 corresponding respectively to the perfect error and the perfect success in the classification, while the intermediate point 0 indicates a classification based on pure chance [47].

In line with what has been mentioned, this paper addresses the design and development of a novel intelligent clinical decision support system applied to the diagnosis of potential obstructive sleep apnea cases. To this end, starting with the information related to the patient’s health profile (anthropometry, habits, comorbidities and drugs taken), and deploying a series of algorithms based on Machine Learning that operate concurrently [49,50,51,52,53], as well as a correcting block based in the joint use of an ANFIS system and a specific heuristic algoritm, the system generates a series of alarms, from which it is possible to estimate whether a patient suffers from the pathology, as well as determine its degree of severity.

Additionally, to assess the performance of the proposed intelligent system, a proof test was carried out on a data set unrelated to that previously used in the training and cross-validation process of the Machine Learning algorithms. In this test datatest, after applying the correction block, AUC values for the ROC curves close to 0.9 are obtained at each of the diagnostic levels with Matthews correlation coefficient values close to 0.6.

This article is organized into five sections. In Section 1, the context in which the proposed system is developed is introduced. Section 2 addresses the conceptual description of the design of the Clinical Decision Support System, highlighting the different stages involved. Once this is done, the implementation of the system is described, pointing to each of the transformations that are applied to the patient’s data until the alerts associated to the AHI levels and the decision-making support are generated. Section 3 presents a demonstration case study that intends to show how the system works. After that, in Section 4 a discussion of the outcomes of the proposed system is presented. Finally, Section 5 contains the main conclusions of this work.

## 2. Materials and Methods

### 2.1. Definition of the System

#### 2.1.1. Database Use

This paper makes use of a data set derived from the analysis and studies performed on 5000 selected patients carried out and registered between the years 2013 and 2022, all of them from a healthcare database belonging to the Respiratory Sleep Disorders Unit of the Pulmonary Department at the Álvaro Cunqueiro Hospital in Vigo (Galicia, Spain). It should be clarified that those cannot be considered as general population, but that all of them are patients who were referred from primary care due to suspicion of a potential OSA case, having undergone a diagnostic sleep test after being screened by expert pneumologists. Said data set includes general and anthropometric information on the patient (sex, age, height, body mass, body mass index, neck circumference length), their habits (smoking and alcohol use), previous illnesses (hypertension, resistant hypertension, ACVA (Acute Cerebrovascular Accident), diabetes, atrial fibrillation, heart failure, COPD (chronic obstructive pulmonary disease), need for home oxygen-therapy, rhinitis and depression) as well as the pharmacological treatments they are receiving (benzodiazepines, antidepressants, neuroleptics, antihistamines, morphic and relaxing/hypnotic drugs). In addition to this, the data set also includes symptoms information coming from a sleep quality-related interview, which was not used in this work as it contains information affected by a high degree of uncertainty.

All those patients underwent sleep tests, cardiorespiratory polygraphies in most cases, determining in each case their apnea-hypopnea index (AHI), a key result both in confirming the disease diagnosis and in determining the severity of the obstructive sleep apnea condition that each patient could suffer.

The entire process of data collection and qualitative assessment was carried out manually by the medical team, except for that information derived from polygraph tests, which is automatically generated by the machine and then collated and incorporated into the database by the medical team in charge.

From the initial data set, 400 patients were randomly extracted, excluding them from the training and validation process of the system, and oriented to carrying out a test of the predictive capabilities of the intelligent clinical decision support system proposed itself. The objective was to carry out the test using an independent data set that was different from those used in the implementation. In this way, it was possible to analyze the outlined proposal, not only from a theoretical viewpoint related to its architecture, but also from a practical perspective, demonstrating its relevance and generalization capabilities with new data, as well as its potential applicability in the field of study [54].

After extracting the 400 patients that would be used in that system test, there was a set of 4600 patients for model training. Table 1 presents a summary of the main descriptors of the data set used.

#### 2.1.2. Conceptual Design and Description of the System

**Figure 1:**
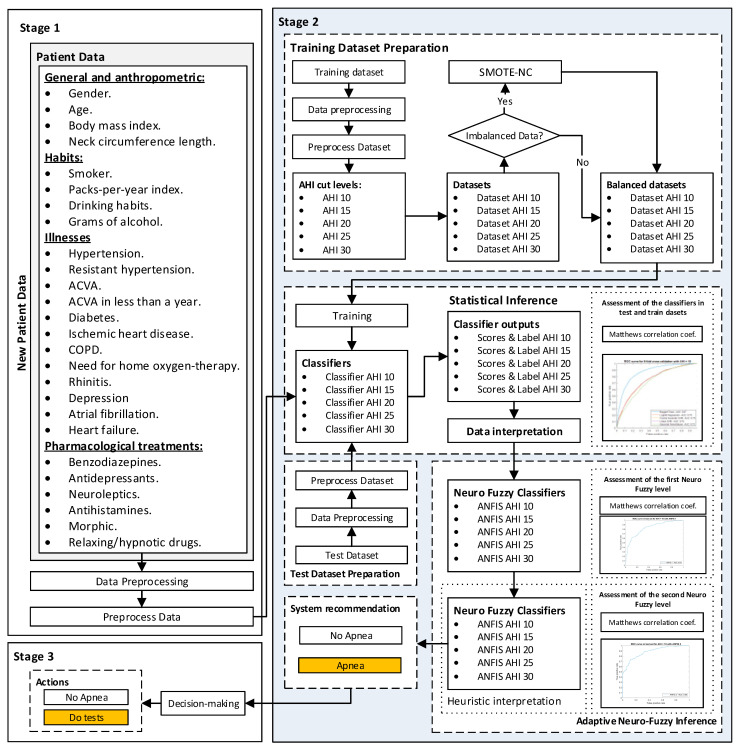
Flowchart of the Intelligent Clinical Decision Support System, showing how the information progresses through three stages. In Stage 1, the initial information is collected and pre-processed. In Stage 2, the processing of the collected information is carried out, and finally, in Stage 3, a recommendation regarding the patient’s diagnosis is provided.

Figure 1 shows the flowchart of the Intelligent Decision Support System (IDSS) proposed in this work, which will be described below. Broadly speaking, the proposed IDSS can be understood as a binary classifier that determines whether the data associated to a certain patient are compatible with the prognosis of them suffering from OSA. It could even perform case analysis tasks by identifying recurrence in undiagnosed or misdiagnosed patients. It is, therefore, an intelligent predictor since it uses inferential models, which help the medical team to decide if the patient is to be diagnosed with the disease, and hence its classification as a clinical decision support system. However, this support only affects one level of information flow, since the true predictive nucleus is the set of inferential techniques that finally allow the implementation of the binary classifier. In this sense, an inferential engine, whether statistical, symbolic or a reasonable combination of both, must always start from a set of explanatory, or independent, variables and another set of explained, or dependent, variables; that is, what is known must be introduced into the inference and what is intended to be predicted must be placed into the output, so that the inference process can establish relationships between both sides of the inference engine (the nature of these relationships is the main difference between the different existing inferential models). When determining and selecting the input explanatory variables, a balance must always be sought between the formalization and diversification capabilities that they have about the problem and the underlying statistical relationships that may exist. It is clear that if the variables have statistically significant relationships, their value in the prediction is not relevant; in the same way that if variables are introduced without apparent causality with the explained variable, optimal results will not be achieved either. In this case, the medical team involved in this work determined that practically the whole set of variables present in the database, identified in Figure 1 and Table 2, Table 3 and Table 4, could be relevant for the prediction even assuming that there are certain factors that are more indicative of a suspected case. This was intended to represent the largest causal chain that could affect the prediction, grouping all the variables and trusting the methodology to determine the final result.

##### Stage 1: Collection of Patient Information

The first stage of the IDSS focuses on the collection of patient information. The information used in this work is generally objective in nature and can be divided into four large groups: general and anthropometric data, habits, diseases suffered, and medications taken by the patient.

The information related to the general and anthropometric data of the patient is summarized in Table 2. All these data may in turn be classified, according to their nature, into numerical data and categorical data.

On the other hand, there is the information related to the patient’s habits, which is summarized in Table 3. As before, the data may be classified as numerical and categorical.

Information is also collected regarding the diseases suffered by the patient, and the pharmacological treatments they receive, which were previously listed in Section 2.1.1 and are summarized in Table 4. Both the data related to the diseases and the treatments that the patient receives are considered as binary categorical; that is, either the pathology in question is suffered or not and, in the same way, either the corresponding drug is supplied or not.

##### Stage 2: Data Processing

Once the patient information has been collected and structured, it is processed by the intelligent clinical decision support system using a series of concurrently operating Machine Learning-based statistical classifiers [49,50,51,52,53]. In order to complete and, to a certain extent, correct and improve the predictive accuracy of these classifiers, the input data set, labeled according to the statistical classifiers, is processed sequentially through two adaptive neuro-fuzzy inference systems (ANFIS) incorporating, in the second, the influence of a specific heuristic algorithm. The definition and configuration of these statistical classifiers initiated from a clinical data set, previously introduced in Section 2.1.1, from which different training data sets are built and labeled with the *apnea* and *non-apnea* classes, according to different AHI threshold levels (10, 15, 20, 25 and 30). It is important to note that depending on the preferences of the medical team and the clinical aspects to be taken into account, it will be possible to modify these thresholds and define as many of them as necessary. After that, the classifiers will be trained, which will make it possible to determine a collection of scores and labels associated to new patients, facilitating the estimation of the severity of the condition. As the system is used, it will be possible to evaluate its performance and correct possible unwanted behaviors. To do this, as mentioned, the system is provided with a correcting mechanism at the output of each of the different statistical classifiers based on the joint and sequential use of two adaptive neuro-fuzzy inference systems (ANFIS) and a specific heuristic algorithm included in the processing of the second one of them. Both techniques work by expanding the predictive capabilities of the results of the previous statistical inferential model, acting at each of the levels, significantly increasing the values of sensitivity, specificity and precision, this last measured using Matthews correlation coefficient (MCC), here understood as a metric of the precision of the binary classifier in the predictions made. In this way, by using symbolic approaches together with other approaches of a purely statistical nature, it is possible to improve the performance of the proposed intelligent decision support system, establishing a series of fuzzy rules that make possible the capture of the underlying knowledge in the database used by the statistical classifiers.

##### Stage 3: Generation of Alerts and Decision-Making

Given data from a new patient, at the exit of each level and after applying the classifier and the correcting approach, a label will be obtained which allows determining if the patient to be studied presents an AHI level above the threshold at each level.

The medical team will be able to select a limit AHI threshold level, so that the system allows it to highlight those patients who could be a potential OSA case, recommending sleep tests to confirm or rule out the diagnosis.

### 2.2. Implementation of the System

The intelligent clinical decision support system proposed and presented in Section 2.1 encompasses a series of stages that range from the collection of patient information to the generation of alerts, through the necessary processing and inference of the information collected. Even if the intelligent system has indeed inferential capacity, in this application the labels determined must not be considered as conclusive, but rather a measure, as precise as possible, of the risk that the patient has of suffering from obstructive sleep apnea. Therefore, the system does not have decision-making capacity, since these labels must be confirmed with new diagnostic tests, as the inferential process does not have all the relevant information. In this case, the intelligent system only acts as support for the clinical decision, improving the process by determining a set of labels that are capable of describing statistically significant underlying relationships between the starting data and the diagnostic category. If the formalization of the data were complete, then the intelligent system could have its own decision-making and diagnostic capacity, this being an issue to be explored in future work.

To implement the intelligent system, a software artifact will be used which, in turn, by observing the recommendations of Hevner et al. [55,56], guarantees its potential integration into any hospital information system. The software will cover all the stages listed in Section 2.1.2 allowing the acquisition of data, its processing and its subsequent classification by means of a set of labels. This software artifact is presented in detail below, explaining with the help of a graphical user interface how the information evolves across the different stages.

For the design and development of the software artifact, MATLAB^©^ (R2021b, MathWorks^©^, Natick, MA, USA) was used together with its App Designer module [57] for the development of the graphical interface, with the Classification Learner module [58] for the training of the supervised learning-based classifiers, and the Fuzzy Logic Toolbox [59] for the implementation of the ANFIS. In addition, it was necessary to use Python (version 3.9.12), together with its Imbalanced-Learn library [60] for performing data augmentation using SMOTE-NC.

**Figure 2:**
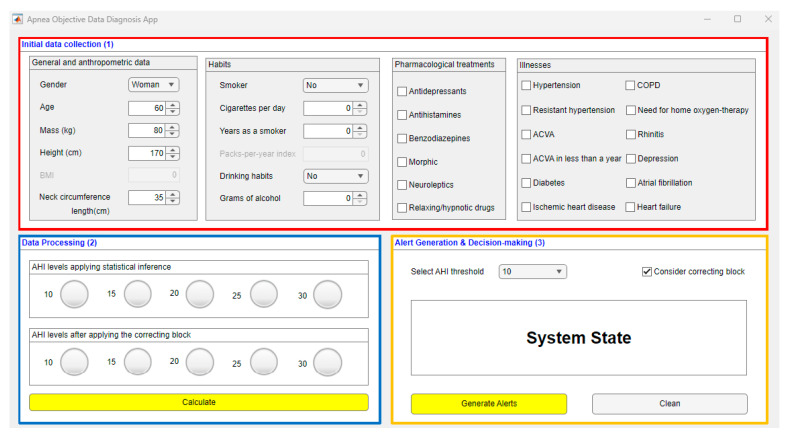
App screenshot. Region (1) refers to the stage of gathering and pre-processing the information of interest about the patient. Region (2) refers to data processing and visualization of AHI levels. Region (3) corresponds to the generation of alerts and decision-making.

Figure 2 shows a screenshot of the graphical user interface of the software artifact. Three main regions may be distinguished in this figure. Region (1) is related to the collection and pre-processing of the information related to the patient. Region (2) represents the data-processing stage and allows visualizing the AHI levels obtained in each of the statistical classifiers, as well as those obtained after the correcting process, using for that a series of indicator lights. Finally, Region (3) allows the user to visualize alerts and generate recommendations.

#### 2.2.1. Data Collection

The patient’s data will be collected through a specific region reserved for this purpose in the software interface. This area is contained in the red box indicated with (1) in Figure 2 and includes a series of questions that the personnel in charge of managing the system must fill in based on the information about the patient to be diagnosed. Filling in this questionnaire in full is crucial and must be carried out unhurriedly and in detail to avoid potential errors or omissions that might lead to an increase in imprecision and vagueness of data, and as a consequence to an increase in the uncertainty of the system.

#### 2.2.2. Data Processing

Once the patient’s data has been entered into the application, they will be processed by the intelligent clinical decision support system. As previously mentioned, a series of statistical classifiers based on Machine Learning are used for this purpose, besides a correcting approach based in the use of ANFIS and a specific heuristic algorithm. The process carried out for the building and definition of these engines is described below. The results obtained in this block are shown by means of a series of colored light indicators, which are comprehended in Region (2) of Figure 2, highlighted with a blue box.

##### Preparation of the Training Dataset

This work starts from a subset of the data set that was previously presented in Section 2.1.1 with 4600 patients. Before carrying out the pre-processing of the data, an analysis of the data was carried out, not finding obvious statistical relationships or significant correlations that suggested eliminating variables from the inferential process. A fraction of these data was of nominal or categorical ordinal [61,62] type, so they were coded using *dummy encoding* (for each variable, a number of auxiliary binary variables are created that replace it, equivalent to the total number of categories in the starting variable minus one). On the other hand, the numerical data (body mass index, age, neck circumference length or grams of alcohol) was scaled in the zero—one range using the Min-Max normalization, whose expression is shown in Equation (1). This type of normalization was chosen since in all the cases contemplated, and at the suggestion of the medical personnel consulted, it was possible to delimit and set maximum and minimum values between which the different variables would move.

(1) $$X^{\prime }=\frac{x_{i}-\min\left(x\right)}{\max\left(x\right)-\min\left(x\right)}$$

Once this was done, the various training data sets were determined. To do this, different AHI threshold levels were established (10, 15, 20, 25 and 30), through which it was possible to classify each patient from the starting data set with the label *apnea* or *non-apnea* depending on whether the AHI level resulting for the patient corresponded to the threshold values set at each level. In this case, if the problem is considered as multivariate [63], the explanatory variables would be the data set that characterizes each patient, while the explained variable is the label assigned to them. This was the way each starting data set was formed, which constitutes a perfectly valid labeled data set to be used in the accurate training process for the application of a Machine Learning algorithm. Thus, different data sets were defined, five in this case, associated with the different AHI threshold levels previously mentioned. Table 5 shows the number of existing cases in each of the levels based on the selected threshold.

Subsequently, the existing imbalance in each of the new data sets between the *apnea* and *non-apnea* labels was analyzed, and if it was the case, an augmentation was made on the data by using a variant of the Synthetic Minority Over-Sampling Technique (SMOTE) [64,65] that allows the management of both numerical and categorical data: the SMOTE for Nominal Continuous (SMOTE-NC) technique. The synthetic data generation strategy with SMOTE-NC used a number of neighbors k = 5, adding data until having 4000 elements in each class for the different data sets. Data augmentation in medical diagnostic and analysis environments is a common practice that tends to improve the results of binary classifiers that essentially underpin any diagnostic predictive approach [52,65].

##### Statistical Inference Algorithms

Once the different training data sets were available, already labeled as *apnea* and *non-apnea* based on the different AHI threshold levels previously established, it was possible to train classifiers based on statistical inference in its Machine Learning sense [66], which are commonly used in Artificial Intelligence and more specifically in the field of Machine Learning, to make predictions with data from new patients.

In order to assess in a practical and massive way the efficiency of the multiple and possible algorithms commonly used in the Machine Learning field, multiple trials were carried out using the MATLAB Classification Learner app [58]. The different algorithms used in the training process are commented in Table 6. A k-fold cross type validation [67] was chosen with k = 5, meaning that the data was initially divided into five equal- or practically equal-size folds. After this, five subsequent training and validation iterations of the algorithm were developed so that in each iteration a different fold was taken for validation while the remaining four were taken for training. Carrying out this validation for different algorithms, five different performance metrics were obtained, in this case for each one of them, thus allowing to choose which one presents the best results.

**Figure 3:**
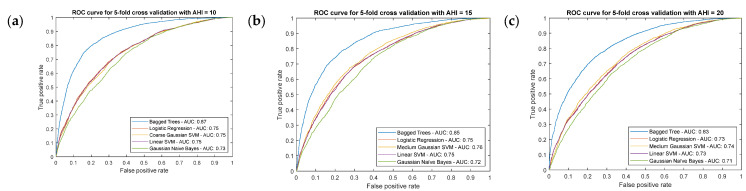

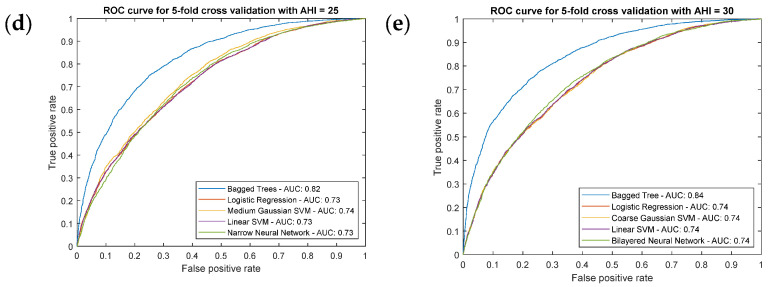
ROC curves for the different algorithms. (**a**) AHI threshold equal to 10; (**b**) AHI threshold equal to 15; (**c**) AHI threshold equal to 20; (**d**) AHI threshold equal to 25; (**e**) AHI threshold equal to 30.

For each AHI threshold, the different models obtained were evaluated through the interpretation of their ROC validation curves taken as a measure of their performance, standing out among them the Bagged Trees algorithm. It is relevant to point out that the selection of one algorithm or another does not lead to any limitation in the system presented, and that in the future, for example in the testing phase of the model, if reasons are observed that justify the replacement of the current calculation algorithms by others, they can be replaced without implying a significant change in the structure of the proposed intelligent system. Figure 3 shows the ROC curves after the validation process and associated with the different mentioned algorithms corresponding to the different threshold levels. In all cases, the Bagged Trees are the approaches that stand out, presenting values of area under the curve between 0.8 and 0.9.

The determination of the algorithm that presents a greater validation level allows performing a first prediction of the risk that a patient has of being assigned to one of the previously established AHI thresholds. However, this is not a conclusive risk, precisely due to the variability inherent in the statistical inferential process. The validation tests only determine the fit of the predictive algorithm to the training data, but in no case do they guarantee success in the prediction of a patient with external and independent data from those used in the training. That is why, in anticipation of the imprecision expected by the set of statistical classifiers, a correct algorithmic approach was designed that sought to improve the classification results by introducing a neuro-symbolic approach and a heuristic adapted to the problem. Both approaches aim to improve the formalization of the problem and capture the underlying knowledge in the prediction.

##### Correcting Approach—Proof Test of the System

In the previous section, *Statistical Inference Algorithms*, the determination of the different statistical classifiers was carried out, obtaining plausible and appropriate results supported by areas under the ROC curve in the cross-validation process between 0.8 and 0.9. At the output of that block, a series of scores and labels were obtained, from which it is now possible to establish a prediction and generate the pertinent alerts.

Although very useful, the classifiers used in the previous stage make predictions through inferential models that, in the vast majority of cases, are opaque or difficult to interpret. Its objective is to fit the input data to some output labels and for this reason its predictions must always be considered under uncertainty; but not only an uncertainty associated with the veracity of the data, but also to the predictive process itself that can force conclusions that are difficult to understand or even contradictory. Explaining the prediction models, however, is essential in medical practice because it not only allows understanding the reasoning process of the system, but also helps to improve its results. In this sense, symbolic inference models, through their formal representation of knowledge, are very useful as long as there are environments and experts with the capacity to formalize the knowledge associated with a certain inferential process. If this is not the case, as happens in the initial diagnosis of apnea, it is possible to implement algorithms that create their own knowledge bases from the existing data and, with this, a set of essential symbolic rules that allow replacing the expert systems that act with complete formalizations of knowledge. Likewise, the use of heuristic algorithms, developed and adapted to the problem, allows to incorporate corrections in the classifier by modeling non-linear and purely stochastic behaviors. These behaviors can be detected through optimization approaches or, as is the case, through comparative analysis of the behavior of the data set with respect to the relationship between the actual and predicted labels. Therefore, to improve the performance and generalization capacity of the IDSS as it is used, the system has been provided with a correcting block based on the sequential use of two ANFIS and a specific heuristic algorithm proposed by the authors. The definition of the correcting block will be improved as the system is used, since it is necessary to know how it behaves with new cases, noting when it succeeds and when it fails. For this, it is necessary to have a history of the outputs of the system, as well as feedback regarding the results of the sleep tests that are performed on patients.

The correcting block was tested through a first implementation that at the same time, allows testing the behavior of the model and selecting those statistical inferential classifiers that present a better performance and generalization capabilities with new data. To this end, 400 patients were reserved that had not been considered in the training process of the system’s statistical classifiers, as commented in Section 2.1.1. In this sense, it should be noted that the statistical classifiers selected after carrying out the proof test do not always coincide with the models that presented a higher AUC in the cross-validation.

To exemplify the implementation of the correcting block, we focused on the threshold AHI = 10. After that, and after evaluating the different patients of the test data set using the different algorithms, we had a relationship of the rates of successes and failures in each threshold level. Based on this information, the correcting block was defined and configured, which was deployed in two levels of action based on the use of two sequential ANFIS. The first one was used to correct the predictive results derived from the statistical inferential block, and the second incorporated a heuristic algorithm in the calculation of some of the ANFIS input variables with the aim of improving the results of the previous step.

To show the use of this corrective approach, the test data was used to exemplify the entire predictive process. With these data, after the evaluations and results obtained, the Logistic Regression algorithm (with an AUC in training of 0.75) was chosen to represent the calculation in the previous statistical inferential section.

*First Level of the Correcting Block—First ANFIS*

Table 7 shows a summary of the configuration of the first ANFIS for the threshold AHI = 10. This first ANFIS is provided with the two scores determined by the classifier, in this case the logistic regression, which precedes it (Score 1 and Score 2), the label predicted by the model (*apnea* or *non-apnea*), and the ‘deviation from Score 1’ value, this understood as the value of Score 1 minus the value of the median of said score in the test data set. The quantitative nature of both Score 1 and its corresponding Score 2 must be taken into account, defined in the case of logistic regression as (1—Score 1) and its correspondence with the binary classifier through an exchange value selected, in this case, by the logistic regression algorithm itself. Similarly, the use of the value called ‘deviation from Score 1′ obeys, on the one hand, to diversify the set of inputs and, on the other, to try to capture the statistical significance that the Score 1 value may have in its input data set. Obviously, factor analyses or other dimensional reduction strategies could be performed to obtain correlational data on the input data. Any other approach could increase, and even improve, the results, leaving these possibilities for future system improvements. The strategy followed to determine the ANFIS parameters was based on applying grid partition and a combined optimization method, based on the use of least-squares and backpropagation gradient descent methods over 10 epochs.

The output of the first level of the correcting block, i.e., the output of the first ANFIS, is expressed as a continuous value after the defuzzification process. This value actually represents membership to the ‘suffering from apnea’ set. Said membership will be conditioned to the choice of a point in the interval of values derived from the ANFIS that indicates the transition between suffering from apnea or not suffering from it, in this case, being assigned to an AHI threshold value of 10. In any case, the first ANFIS determines what could be understood as an apnea risk metric. Said risk, called the ANFIS 1 Score, will be one of the input variables of the second correcting level.

*Second Level of the Correcting Block—Second ANFIS with Heuristic Algorithm*

Starting from the ANFIS 1 Score, the deviation from that ANFIS 1 Score was determined for each case, understood as the value of the ANFIS 1 Score minus the median value of ANFIS 1 in the test data set. This new score, its deviation, as well as the variables that were provided at the input of the first ANFIS (Score 1, Score 2, Predicted Label and the deviation from Score 1) were provided to the second level of the correction block together with a correction factor calculated through the proposed heuristic algorithm. The reasons for choosing the derived scores have already been argued before, making it evident that any other statistical representation variables could be used from the initial data. Regarding the correcting factor, it was based on the calculation of a heuristic algorithm developed for this work and adapted to the prediction data set. Equation (2) shows the mathematical expression of said algorithm.

(2) $$\begin{array}{c} Correction\;factor=Predicted\;Label\\ IF\;log_{10}\left(deviation\;Score\;ANFIS\;1\right)\in \left[-1,-0.645\right]\;AND\;\left|deviation\;Score\;1-deviation\;score\;ANFIS\;1\right|\\ \in \left[0,\;0.8\right]\;\Rightarrow \;correction\;factor=\left|correction\;factor-1\right| \end{array}$$

The definition of the heuristic algorithm is not trivial. It starts with the analysis of the behavior of the data against the registered real and predicted labels, specifying in those variables derived from the behavior with respect to the median for both Score 1 and ANFIS Score 1. Both the distribution of the data and their possible correlations with the labels were exhaustively analyzed and the algorithm expressed in Equation (2) was determined after a search and comparison process. It should be understood that both Score 1 and ANFIS Score 1 actually represent a probability of belonging to the ‘suffering from apnea’ set, exemplifying a metric of a probable risk of a patient suffering from the disease. Therefore, when considering the behavior of these scores based on their median, this is intended to capture that value which represents in a more usual and typical way the relationship between the scores and the real and predicted labels. Taking this as a foundation, it is feasible to then trust in finding a stochastic description of the relationship of the score deviations with the real apnea/non-apnea labels and, with this, to model a factor that adequately corrects the incorrect predicted labels. Now, this presents an immediate limitation associated with the very concept of median. By characterizing a data set in a general way, identifying the value that separates the set into halves and avoiding the bias of the extremes, the correcting factor will improve its operation in the central values of the set, being ineffective in extreme values or with behaviors far from the median. Hence the numerical values that appear in Equation (2) and that fit within closed intervals the deviation of scores.

Once the input variables were established, we moved on to the definition and use of the second ANFIS, the one that works after the application of the heuristic algorithm. Said algorithm, as just mentioned, is provided, as inputs, with the values of: Score 1, Score 2, Predicted Label, Score 1 deviation, Score ANFIS 1, Score ANFIS 1 deviation and Correction Factor.

Table 8 shows the configuration of the second ANFIS for the AHI = 10 threshold. The strategy followed to adjust it is similar to the one followed in the first ANFIS, applying grid partition and a hybrid optimization method, based on the use of least-squares and backpropagation gradient descent methods over 10 epochs.

Once the second ANFIS was obtained, as in the first case, a new score was obtained at its output, the so-called ANFIS 2 score. As already mentioned, the interpretation of the classification derived from this value is subject to establishing a threshold value that marks the separation between scores labeled as ‘apnea’ and those labeled as ‘non-apnea’. To do this, one must start from the set of scores obtained and find, based on the selected threshold value, which of these scores (remember that the ANFIS 2 score will lie between 0 and 1, as the threshold also does) maximizes the results of the binary classifier. With this objective, an iterative optimization process was carried out that calculates, once the final scores derived from ANFIS 2 are obtained, the value of the Matthews correlation coefficient considering different threshold values. That is, for example: a threshold value of 0 is taken and the ANFIS 2 scores are labeled with it. In this case, all the labels would be of ‘apnea’. The Matthews correlation coefficient was calculated for that case according to Equation (3), obtaining a value of 0.1961. The acronyms in Equation (3) were previously explained in Section 1: TN refers to True Negatives, FN refers to False Negatives, TP to True positive and FP to False Positives.

(3) $$Mcc=\frac{TN\cdot TP-FN\cdot FP}{\sqrt{\left(TP+FP\right)\cdot \left(TP+FN\right)\cdot \left(TN+FP\right)\cdot \left(TN+FN\right)}}$$

**Figure 4:**
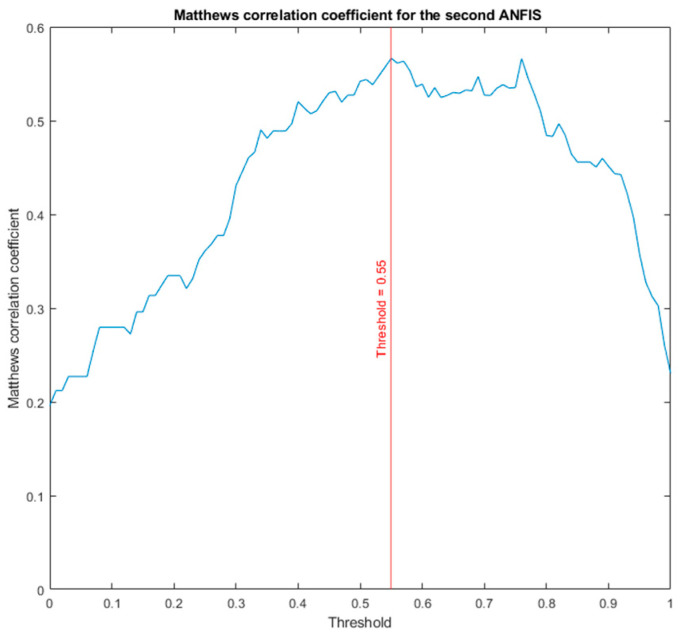
Determination of the optimal threshold for the second ANFIS for AHI = 10.

The process continues, in this case for a threshold value of 0.01, obtaining a coefficient value of 0.2121. Thus, with threshold steps of 0.01 until reaching one. The graph that includes this optimization can be reviewed in Figure 4, where it can be seen that the threshold value that optimizes the correlation coefficient is 0.55. This process, in reality, has no inferential or predictive value, but corrects and, in a way, delimits the influence of a decision on the accuracy of the binary classifier. Choosing one threshold or another, since this choice is not conditioned or automated based on a confusion matrix, as is the case, introduces an uncertainty bias in the classifier’s own evaluation. When going through all the options, we could not identify the best one of them because, in reality, the scores derived from ANFIS 2 do not change, but rather we identified that point that allowed us to transform the scores into binary labels of apnea and non-apnea more efficiently.

**Figure 5:**
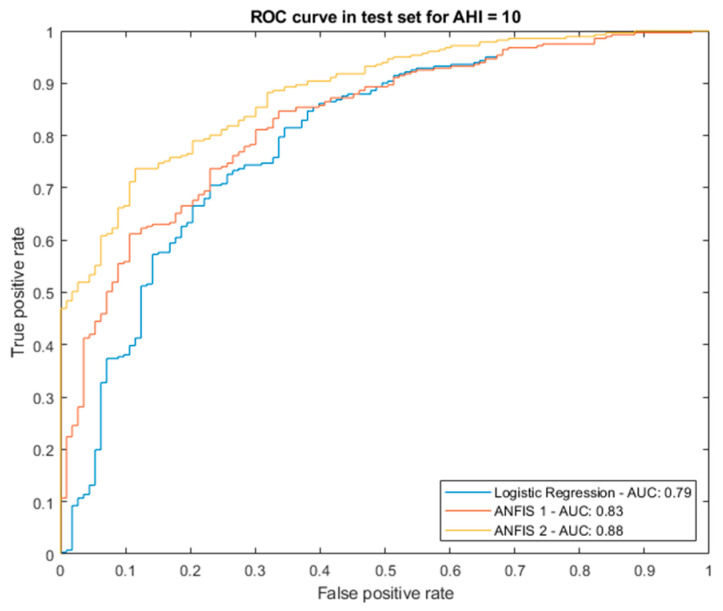
Comparison of areas under the curve for AHI = 10 after applying the correcting approach.

Once the correcting levels were completed and the optimal threshold was identified, it was possible to study the results of the system’s proof test. Figure 5 shows the ROC curves on the test data set for the Logistic Regression model, as well as the ROC curves associated with the two levels of the correcting block (at the output of ANFIS 1 and ANFIS 2, respectively), highlighting the clear and considerable associated improvement achieved, moving from an AUC value of 0.79 to 0.83 in the first correcting level, and from 0.83 to 0.88 after the application of the second correcting level.

*Proof test results*

The procedure for determining and calculating the rest of the correcting blocks corresponding to the other AHI levels is similar to that commented for AHI = 10. Table 9, Table 10, Table 11, Table 12 and Table 13 below show a summary of the results obtained on the test data set for the different AHI levels in terms of AUC and MCC. In the proof test, the binary classifier, after passing through the sequential cycle of the ANFIS and the heuristic algorithm, AUC of the ROC curves close to 0.9 were obtained in each of the diagnostic levels with Matthews correlation coefficients close to 0.6. It must be taken into account that the test data were not previously analyzed, so there is a conviction that many of them introduced an epistemological uncertainty in the prediction, forming labeled lines that respond more to pure chance than to logical behavior. Considering this, the results obtained in the proof test were truly significant.

The results described in previous Table 9, Table 10, Table 11, Table 12 and Table 13 show, on the one hand, that the correcting approach was appropriate and fit for the prediction of apnea and, on the other, that the intelligent decision support system achieved performance levels significantly above those of other more common approaches already discussed in Section 1. One can also observe the robustness of the heuristic algorithm developed, since the precision percentage clearly increased at all AHI levels.

Having clarified this, the last step in testing the system involved only selecting the best prediction algorithm once results were obtained for all of them, applying the correcting approach and taking into account the different AHI levels that this system introduces. Thus, once the results obtained in the test set have been analyzed, for each AHI level the classifier that presents the best and most appropriate results in terms of AUC and MCC at the output of the correcting block must be selected. After analyzing the data corresponding to the proof test, it was determined that the Logistic Regression for AHI = 10, the Medium Gaussian SVM for AHI = 15, 20 and 25, and the Coarse Gaussian SVM for AHI = 30 were the most accurate algorithms. Table 14 presents a summary of the sensitivity and specificity values obtained by the IDSS at the output of the correcting block for each of the selected algorithms.

#### 2.2.3. Generation of Alerts and Decision Making

Once the entire process has been completed, the system returns a label (‘apnea’ or ‘non-apnea’) for each AHI level.

The medical team will then be able to decide whether or not to apply the correction, and will establish an AHI level above which the system will generate alerts.

The results obtained in this block are shown in Region (3) of Figure 2, highlighted with an orange box.

## 3. Case study

This section presents a case study for the intelligent clinical decision support system proposed in Section 2, aiming to illustrate its operation in detail. Although this article is presented as a proof of concept, a test data set was reserved to visualize the usefulness and validity of the model in a data set unrelated to that one used for model training. Thus, the data included in the example belongs to the new test data set and was neither used for training nor validation.

As can be seen, this case study is aimed to exemplify its potential use as a diagnostic tool in sleep-related respiratory disease diagnostic units and serve, incidentally, as a proof of concept.

### 3.1. Compilation of Patient Information

Table 15 summarizes the data about the patient to be studied, who, after performing sleep studies, was diagnosed with an AHI of 3.70. The patient was referred from primary care due to a suspected OSA case because her husband indicated that she had sleep apnea. In addition, they indicated that she was a high-intensity snorer and felt tired on a daily basis. The patient’s data was not included in the data set used for the training and validation of the inferential statistical part of the system.

The objective of this example was to verify that the system is mainly capable first, of determining if a patient suffers from apnea, and second, if that is the case, of identifying to which possible AHI level they can be assigned.

Once the data was entered into the application, it was processed by the intelligent clinical decision support system.

### 3.2. Data Processing

After entering the data into the application, it was processed by the different previously mentioned statistical classifiers, as well as by the correcting block, linked to different AHI levels.

**Figure 6:**
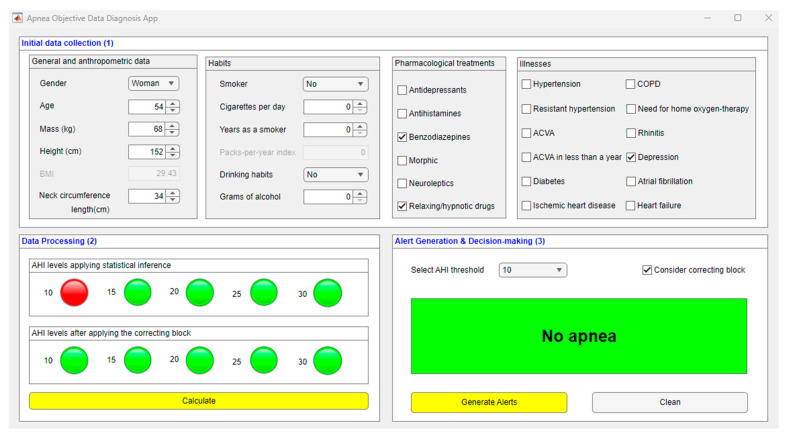
Application screenshot. Determination of the risk of suffering from OSA associated to the different threshold levels.

Figure 6 shows a screenshot of the application in which the AHI levels obtained can be observed. In the box related to the statistical inference, a colored in red light indicator is shown for an AHI level of 10; that is, a priori the patient would present an AHI ϵ [10, 15). Below that box, another box related to the model corrections is shown in which, in this case, the model prediction is corrected, as can be seen in the colored in green indicator light; that is, the patient would present an AHI ϵ [0, 10), or in other words, the patient does not suffer from OSA.

In this case, the patient was referred to the hospital where she underwent a polygraph that determined an AHI level of 3.70, which corresponds to the threshold assigned by the system.

### 3.3. Generation of Alerts and Decision-Making

Once the calculation of the AHI levels has been carried out, the medical team must establish the limit AHI level to evaluate the patient, and from this, consider whether confirmatory sleep tests should be performed. In this example case, the system determined that the patient did not suffer from apnea, thus recommending the medical team to not subject her to further diagnostic tests. Even so, the system only supports the final decision, which is always the exclusive responsibility of the medical team. In the actual case, the patient was referred to polygraphic diagnostic tests that confirmed the results predicted by the system.

The aim is not to identify the specific AHI value, a measure derived from subsequent diagnostic and confirmatory studies, but rather to point out in advance and non-invasively the patient’s risk of the disease. With this test, supported by the intelligent system, symptoms of apnea in patients could be predicted preventively, its severity classified according to the previously established AHI thresholds and, all in all, optimizing the costs and inconvenience of subsequent unnecessary tests.

### 3.4. Expansion of the Results

With the aim of expanding the case study, new examples were carried out following a process similar to that discussed in Section 3, as can be seen in Table 16. All the cases presented were referred from primary care due to a suspicion of being potential OSA cases, and were accompanied by the AHI value obtained after carrying out the corresponding sleep test.

From a direct analysis of the 20 cases listed in Table 16, all of them corresponding to the proof test and included in this case study, the system’s prediction was verified correct in all cases and, in addition, the correcting approach corrected and improved predictions derived from simple statistical inference. All of this suggests that the system significantly benefits from the inclusion of the correcting approach, which not only improves the data quantitatively, but also provides the predictive system, as is discussed below in the Discussion, with better performance and better logical functionality.

## 4. Discussion

The OSA is a pathology that has a high impact on the health and well-being of patients. The usual approach for its diagnosis is based on sleep tests, cardiorespiratory polygraphies or polysomnographies. These are laborious and expensive tests that are not suitable for mass screening of the general population. For these reasons, at this time, and considering the degree of development of predictive and classification algorithms within the field of artificial intelligence, the need and possibility of developing intelligent tools that support decision-making, while improving diagnostic processes, are being contemplated more than ever.

This work proposed a novel intelligent decision-support system applied to the diagnosis of OSA that deploys a series of statistical classifiers for different threshold AHI levels, complemented by a correcting approach based on the joint and sequential use of two ANFIS, with a particular heuristic model used in the second ANFIS. Analysis of the results at each level allows the medical team to establish a metric for the patient’s risk of suffering from apnea, including making an estimation for the AHI value.

The presented intelligent system can, and should, be understood as a binary classifier having predictive capabilities through the representation of a complex inferential process, followed first by the statistical algorithms and then by the two ANFIS and the heuristic algorithm. In this correcting block, the model incorporates an approach that combines neural networks with fuzzy logic, which makes it possible to incorporate, albeit indirectly, the benefits of symbolic inference, especially in relation to the formalization of knowledge, together with the development of a heuristic algorithm that allows to optimize the problem and to increase the success in the prediction. This increased capacity for reasoning and information extrapolation allows the system to be given a primordial inferential capability supported by its inherent capability for optimization and representativeness of the data. That is, the intelligent system can establish inherent, and perhaps—a priori—difficult to explain reasonings about the relationships between the data and the presence of apnea, thus reducing the large dimensionality of the problem. Of course, in medical applications, especially diagnostic ones, the traceability and explanation of automatic reasoning processes become even more important. Statistical inferences are not always obvious and may even lead to false conclusions. However, in cases such as the diagnosis of apnea, it is not always possible to have an argued, coherent and truthful database on which to base logical symbolic inferences. Thus, in this conceptualization of the system, and because of the absence of a complete formalization of the knowledge required to make the prediction, priority is given to its capability for representing this data; that is, for combining these relationships and providing an objective and graphic metric on which the medical team can support its final decision. In this way, the medical team will be able to use the system, integrating it into a larger clinical decision support system, also incidentally delimiting part of the uncertainty that is inherent to the decision to be made.

In this sense, a diagnosis process can be considered as a decision under uncertainty conditions; this uncertainty can, in turn, be expressed in different ways: lack of knowledge, multiplicity and multivariety of data, ambiguity, inaccuracy, etc. To delimit uncertainty, probabilistic and non-probabilistic approaches are used in which the inferential results indicate probabilities or degrees of belonging to a specific diagnostic label. Controlling all uncertainty sources is a very complex task that is beyond the objectives of this work, but of course, its presence must be acknowledged and contemplated in this system. In the system presented, uncertainty is reduced through an approach that combines, on the one hand, a reduction in the imprecision and vagueness of the starting data, obtained from the answers to a specificly elaborated diagnostic questionnaire and, on the other, using several classification algorithms together with neuro-symbolic models and heuristic approaches which, as a whole and using different inference mechanisms, are capable of establishing percentages of belonging to different AHI diagnostic levels. 

The consistency of the inferential union, or what it is, the justification of joining and defining a sequential architecture of classification algorithms, is a non-trivial process that should, in our opinion, be diverted towards the analysis of data formalization and the guarantee that no relevant information is lost in the chain of causality by using unexplained formalizations such as those used in purely statistical inferential models. When formalizing, the information of a problem is expressed by usual logical entities, for example IF…THEN rules or, in any case, through numbers with no apparent explanation. This is where the real challenge lies: knowing which numbers contain the necessary information to make the prediction without being able to express this information symbolically. For this reason, formalization based on numerical data must be as general as possible, avoiding spurious modifications and sources of uncertainty added by means of non-recommended statistical operations.

In addition, the use of large data sets and class-balancing algorithms in binary classifiers such as the one described guarantees, on the one hand, an increase in the representativeness of all possible casuistries and, on the other, a trend towards normality in case distributions, always a favorable matter in inferential statistical approaches. If we also add to this the suggestion of developing different classifiers based on different AHI levels, together with a correcting approach that significantly increases the success of the classification, a trend is achieved towards—a partial, that is true—formalization on the part of knowledge, which constitutes the foundation—together with the diversification—of expert systems whose formal analogy with learning algorithms makes it possible to guarantee the reduction in epistemological uncertainty, which is without a doubt the most difficult type to control.

Thus, considering the conceptualization of the intelligent system, its performance must be evaluated, not only from the point of view of the results, whose comparison will be discussed later, but fundamentally its coherence in the ability to represent knowledge must be analyzed. It has already been shown that the system is capable of making a first statistical inference that, although indefinite and little known, allows plausible predictions to be made. Likewise, it incorporates a corrective approach made up of two sequential ANFIS and a heuristic algorithm that work together with the second of them. Could it be said that the data set, the symbolic rules of ANFIS and the heuristics themselves make up an argumentative and ontological knowledge base? In reality, this would not be the case, since the knowledge base must be structured, defined, explained and related in such a way that it constitutes a set of validated and permanent statements. Lacking this knowledge, inferential models inevitably tend towards statistics, since it is no longer possible to infer from knowledge, but only from data, that is, unstructured, indefinite, confused, unrelated and ephemeral information. Machine learning algorithms take this data and build mathematical fantasies that relate that data to some labels, but being fantasies, they are often ideal constructs whose coherence does not transcend beyond the scope of action of the algorithm itself. How then to act in the absence of a knowledge base? In this work, a strategy was followed that tried to capture knowledge in a deferred way, enriching the data. Although the first statistical classifiers behave in the traditional way, the ANFIS already works it in a different way. By introducing a data set into these neuro-symbolic models, namely the outputs of the previous algorithms and the labels, the numerical data is fuzzified and acquires additional value, starting to behave as a symbol. The data no longer only represents a metric or a value, but also represents a prediction and, in a way, a relationship, certainly stochastic, but a relationship nonetheless that allows, together with all those data, to represent a true fact without the need for syllogisms. If, in addition, the ANFIS are linked, then the second one is capable of partially formalizing a very basic and heterogeneous knowledge base that helps to improve reasoning. The heuristics used contribute to this, yes, in a determined way. This, by definition, is the ability to generate knowledge and increase it, so identifying an algorithm that is capable of modeling the underlying knowledge as a series of numbers is an ideal complement to a fuzzy inferential model such as ANFIS. Therefore, it is possible to affirm that a prediction problem treated from the absence of a complete formalization of knowledge and of the implications and relations of the variables that make up the database may be capable to carry out classifications that are certain and truthful, consistent, and in a way, permanent and explainable. 

In addition to the advantages in terms of data processing and the reduction of the uncertainty associated with the use of intelligent systems, in this case there are obvious and clear improvements to diagnosis and healthcare processes. The determination of suffering from apnea associated to different AHI thresholds is a very valuable metric when comparing different patients suspected of suffering OSA, since it facilitates the subsequent prioritization and generation of waiting lists for confirmatory diagnostic tests, therefore reducing the inherent subjectivity associated with the assessment carried out by the medical team.

On the other hand, the choice for using part of the information that is present in the electronic health records, which is valid in itself, could be an important advantage. This would facilitate the making of predictions based on the information available in those, without the need for in-person meetings with the patients in long questioning sessions, reducing the uncertainty from interaction, as well as potential falsehoods, omissions or errors in data collection. This opens a path for future development and research since, if the necessary updated information were available, OSA population screening campaigns could be deployed gradually and progressively, thus alerting those patients who present potential risk cases.

Additionally, this tool could be very useful for physicians who are not specialized in apnea diagnosis, such as those dedicated to primary care. Through this system, they could initially analyze the risk level that a patient suspected of suffering from obstructive sleep apnea may have, assessing, based on the results obtained, whether they should be referred to specialized consultations for study. Of course, this is made possible thanks to the system’s ability to formalize knowledge, as argued before, which makes it in part possible to compensate for the lack of specific knowledge of the non-specialist doctor with the formalization that the system itself proposes.

Of course, taking into account that the formalization is incomplete, in no case should the medical opinion and decision be ignored. In the same way, and for similar reasons, the tool also has its place in hospital units specialized in sleep-disordered breathing, since it can help determine the severity of the pathology by the interpretation of the different AHI threshold levels. It could also be extremely useful in the detection and identification of undiagnosed cases in a group of patients. The presented binary classifier can not only determine apnea cases, but also guess undiagnosed cases by analyzing accessible data records.

All of the uses previously mentioned are of great help to medical teams and, without a doubt, could have a very relevant impact on the management of hospital resources. The number of patients referred to specialized sleep units could be reduced by discarding those who do not really present a diagnosis compatible with OSA, with consequent saving of resources and time. The quality of the services provided could also be improved, focusing only on those suspected patients, and prioritizing tests and treatment of the most serious cases from the outset.

### Relevance in the Field of Study

The results obtained in the proof test are truly significant, with AUC values lying between 0.8 and 0.9, and MCC close to 0.6. In general, it is reasonable to claim that these results are an improvement over similar works in the diagnosis of OSA. In addition to this, the results obtained are superior to those of the questionnaires traditionally used in this field (Berlin Questionnaire, STOP-Bang, STOP, which usually present a high sensitivity at the cost of a low specificity), as well as, in general, the rest of the approaches reviewed in Section 1 that apply artificial intelligence techniques.

On the other hand, the strategy followed in the design of the system that involves using different inference models concurrently determining a membership to different thresholds of AHI [49,50,51,52] is also a novelty in the field of study, since in the previous works analyzed it is observed that the authors establish a fixed AHI threshold level (usually 5 or 10) [16,17,19] only, which could mean recommending a sleep test to patients who do not need it, thus overdiagnosing them. An exception to that is the work by Wen-Chi et al. [20], in which different models are analyzed for AHI threshold levels equal to 5, 15 and 30.

After discussing the benefits of the proposed system, and demonstrating its predictive capabilities in the diagnosis of OSA, its relevance is analyzed in Table 17 below from a technical point of view in comparison with other proposals, considering for this purpose a series of criteria that are usual in the field of design and development of intelligent systems [52,53,68]:

- Internal architecture: aiming to analyze the reliability of the model from the point of view of its ability to manage uncertainty.
- Scalability: aiming to determine the model’s ability to add or remove blocks from the system.
- Inference: aiming to analyze the system’s ability to use symbolic reasoning supported by the complete formalization of a knowledge base.
- Learning: aiming to assess the system’s ability to incorporate learning approaches that are common in the field of Machine Learning.

After analyzing the contents of Table 17, in general terms, it can be concluded that the approaches commonly used in the field of OSA detection are simple, and mainly apply statistical inferential approaches through the use of algorithms commonly used in the field of Machine Learning. The proposed system, in its first layer of action, also uses inferential statistical approaches that are later enriched and improved by adding the correcting block, which, through the use of ANFIS and a specific heuristic algorithm, allows carrying out a better and more efficient management of knowledge and information, reducing and delimiting the associated uncertainty. This undoubtedly represents a clear novelty in the field of study of the OSA. All that has resulted in clearly remarkable test results that corroborate the assumptions made in the conceptual development of the system.

## 5. Conclusions

In this work, a new intelligent system applied to the preventive and early diagnosis of patients presenting potential sleep apnea symptoms was presented. It follows an approach based on the combined use of Machine Learning algorithms together with ANFIS and heuristic models to determine if a certain patient suffers from apnea, associating such prediction to a series of AHI threshold levels. The incorporation of the corrective approach allows to improve the predictive accuracy of the system as it is being used. Its main novelty, therefore, from the clinical point of view, lies in determining the presence of the disease based on an initial data set, which allows the medical team to carry out a prior assessment of the patient, speeding up the diagnostic processes and reducing the need for confirmatory tests. Likewise, it reduces the dimensionality of the problem and allows, through the second ANFIS, to have certain logical values that explain the diagnostic reasoning. From a technical point of view, the intelligent system possesses an indirect capacity to formalize knowledge, which is unusual in pure statistical approaches, thus allowing to improve its predictive capacity, as its proof tests certify.

The performance of the proposed system was analyzed using a test data set with 400 patients, unrelated to those used in the construction of the model, yielding AUC values in the range 0.8–0.9, as well as Matthews correlation coefficients close to 0.6. Despite the results obtained and their associated clinical relevance, in the future it will be necessary to carry out more new tests using data from other hospitals, which will allow confirmation of the results obtained in the tests carried out, thus consolidating the proposed system.

Likewise, evident improvements, such as the presence of a complete formalization of the diagnostic problem, contemplating the symptoms evidenced by the patient, the incorporation of expert systems, the modeling of symbolic knowledge bases and the use of hybrid approaches, could improve not so much the results as the reliability of the system.

## Figures and Tables

**Table 1:** Summary of the main descriptors for the training data set.

Descriptor	N (%)/Mean ± SD	
	Male	Female
Number of patients	2930 (63.70%)	1670 (36.30%)
Age	55.16 ± 13.32	55.12 ± 13.68
BMI	31.62 ± 5.68	34.91 ± 8.47
Neck perimeter	42.72 ± 3.86	38.11 ± 4.00
Hypertension	709 (24.20%)	360 (21.56%)
Resistant hypertension	17 (0.58%)	8 (0.48%)
ACVA	29 (0.99%)	6 (0.36%)
ACVA less than a year before	10 (0.34%)	1 (0.06%)
Diabetes	207 (7.06%)	119 (7.13%)
Ischemic heart disease	112 (3.82%)	21 (1.26%)
COPD	38 (1.30%)	12 (0.72%)
Need for home oxygen-therapy	5 (0.17%)	5 (0.30%)
Rhinitis	75 (2.56%)	44 (2.63%)
Depression	98 (3.34%)	158 (9.46%)
Atrial fibrillation	82 (2.80%)	36 (2.16%)
Heart failure	34 (1.16%)	16 (0.96%)
Benzodiazepines	89 (3.04%)	111 (6.65%)
Antidepressants	78 (2.66%)	115 (6.89%)
Neuroleptics	14 (0.48%)	5 (0.30%)
Antihistamines	5 (0.17%)	10 (0.60%)
Morphic	4 (0.14%)	5 (0.30%)
Relaxing/hypnotic drugs	143 (4.88%)	183 (10.96%)

**Table 2:** Information related to general and anthropometric data of the patient.

Data	Data Type	Comments
Gender	Categorical	Male/Female
Age	Numerical	-
Height	Numerical	It is not fed to the algorithm, but only used for BMI calculation
Body mass	Numerical	It is not fed to the algorithm, but only used for BMI calculation
Body mass index (BMI)	Numerical	Derived datum, calculated from height and body mass
Neck circumference length (NCL)	Numerical	-

**Table 3:** Information related to data habits of the patient.

Data	Data Type	Comments
Smoker	Categorical	Yes/No/Former smoker
Cigarettes per day	Numerical	It is not fed to the algorithm, but only used for packs-per-year index calculation
Years as a smoker	Numerical	It is not fed to the algorithm, but only used for packs-per-year index calculation
Packs-per-year index	Numerical	Derived datum, calculated from cigarettes per day and years as a smoker
Drinking habits	Categorical	Yes/No/Casual
Grams of alcohol	Numerical	Grams of alcohol per day

**Table 4:** Information related to illnesses and pharmacological treatments.

Illnesses		
Data	Data type	Comments
Hypertension	Categorical	Yes/No
Resistant hypertension	Categorical	Yes/No
ACVA	Categorical	Yes/No
ACVA in less than a year	Categorical	Yes/No
Diabetes	Categorical	Yes/No
Ischemic heart disease	Categorical	Yes/No
COPD	Categorical	Yes/No
Need for home oxygen-therapy	Categorical	Yes/No
Rhinitis	Categorical	Yes/No
Depression	Categorical	Yes/No
Atrial fibrillation	Categorical	Yes/No
Heart failure	Categorical	Yes/No
**Pharmacological treatments**		
**Data**	**Data type**	**Comments**
Benzodiazepines	Categorical	Yes/No
Antidepressants	Categorical	Yes/No
Neuroleptics	Categorical	Yes/No
Antihistamines	Categorical	Yes/No
Morphic	Categorical	Yes/No
Relaxing/hypnotic drugs	Categorical	Yes/No

**Table 5:** Summary of the number of cases according to the different threshold levels.

AHI = 10 Dataset		
AHI < 10	AHI ≥ 10	Total
1261	3339	4600
**AHI = 15 Dataset**		
AHI < 15	AHI ≥ 15	Total
1773	2827	4600
**AHI = 20 Dataset**		
AHI < 20	AHI ≥ 20	Total
2240	2360	4600
**AHI = 25 Dataset**		
AHI < 25	AHI ≥ 25	Total
2603	1997	4600
**AHI = 30 Dataset**		
AHI < 30	AHI ≥ 30	Total
2907	1693	4600

**Table 6:** Machine Learning models used in the Classification Learner App.

Model Type	Variants
Decision Trees	Fine, Medium, and Coarse Tree models
Logistic Regression	-
Naïve Bayes	Gaussian and Kernel Naïve Bayes
Support Vector Machines	Linear, Quadratic, Cubic, Fine Gaussian, Medium Gaussian, and Coarse Gaussian Support Vector Machine
Ensembles	Bagged Trees and RUSBoosted Trees
Neural Networks	Narrow, Medium, Wide, Two-layer, and Three-layer Neural Networks

**Table 7:** Implementation of the first ANFIS for AHI = 10.

ANFIS 1		
Input Data	Range	Output Data
Score 1 (μ_1_)	0–1	Prediction
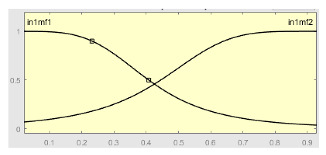		*mf*_1_ = −17.24 · μ_1_ + 37.24 · μ_2_ + 0.87 · μ_3_ − 19.78 · μ_4_ + 20 *mf*_2_ = 4.90 · μ_1_ − 15.37 · μ_2_ − 3.96 · μ_3_ + 9.71 · μ_4_ − 10.47 *mf*_3_ = −26.76 · μ_1_ + 43.10 · μ_2_ + 15.22 · μ_3_ + 78.11 · μ_4_ + 16.34 *mf*_4_ = −31.48 · μ_1_ − 38 − 35 · μ_2_ − 69.45 · μ_3_ + 2.06 · μ_4_ − 69.83 *mf*_5_ = 10.29 · μ_1_ − 60.59 · μ_2_ − 1.13 · μ_3_ + 38.80 · μ_4_ − 50.30 *mf*_6_ = −3.47 · μ_1_ − 7.01 · μ_2_ + 1.64 · μ_3_ − 2.84 · μ_4_ − 10.48 *mf*_7_ = −81.79 · μ_1_ + 59.12 · μ_2_ − 19.80 · μ_3_ + 33.90 · μ_4_ − 26.67 *mf*_8_ = 48.23 · μ_1_ − 20.29 · μ_2_ + 28.65 · μ_3_ − 35.24 · μ_4_ + 27.94 *mf*_9_ = 12.99 · μ_1_ − 19.31 · μ_2_ − 0.36 · μ_3_ + 7.85 · μ_4_ − 6.32 *mf*_10_ = 0.01 · μ_1_ + 2.87 · μ_2_ − 1.54 · μ_3_ − 1.46 · μ_4_ + 2.97 *mf*_11_ = 97.50 · μ_1_ − 104.10 · μ_2_ − 6.24 · μ_3_ − 39.17 · μ_4_ − 6.59 *mf*_12_ = −15.13 · μ_1_ − 11.65 · μ_2_ − 27.04 · μ_3_ − 7.19 · μ_4_ − 26.79 *mf*_13_ = −17.24 · μ_1_ + 37.24 · μ_2_ + 0.87 · μ_3_ − 19.79 · μ_4_ + 20 *mf*_14_ = −4.89 · μ_1_ − 15.37 · μ_2_ − 3.96 · μ_3_ + 9.71 · μ_4_ − 10.47 *mf*_15_ = −25.76 · μ_1_ + 43.10 · μ_2_ + 15.22 · μ_3_ + 78.11 · μ_4_ + 16.34 *mf*_16_ = −31.48 · μ_1_ − 38.35 · μ_2_ − 69.45 · μ_3_ + 2.06 · μ_4_ − 69.83
Score 2 (μ_2_)	0–1	
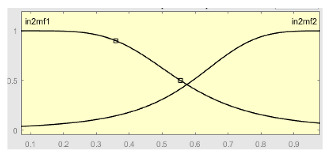		
Predicted Label (μ_3_)	0–1	**Initial configuration**
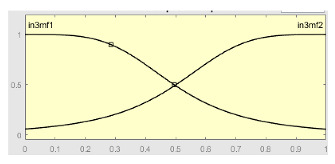		Fuzzy structure: Sugeno-type. Antecedents membership function type: bell-shaped. Consequents membership function type: linear. And method: PROD. Or method: MAX. Implication method: MIN. Aggregation method: MAX. Deffuzification method: Weighted average of all rule outputs. Number of fuzzy rules: 16.
Score 1 deviation (μ4)	0–0.48	
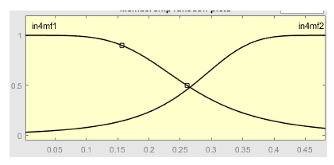		
**Summary of rules**		
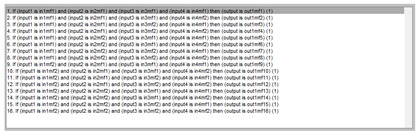		

**Table 8:** Implementation of the second ANFIS for AHI = 10.

ANFIS 2		
Input Data	Range	Output Data
Score 1 (μ_1_)	0–1	Subset of 128 output mf’s
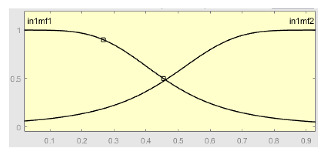		*mf*_1_ = 1.05 · μ_1_ − 2.79 · μ_2_ − 1.08 · μ_3_ + 1.84 · μ_4_ + 52.66 · μ_5_ − 54.87 · μ_6_ + 0.12 · μ_7_ − 1.74 *mf*_2_ = 1.21 · μ_1_ + 0.74; μ_2_ − 1.13 · μ_3_ − 1.47 · μ_4_ + 10.89 · μ_5_ − 7.08 · μ_6_ + 2.06 · μ_7_ + 1.95 *mf*_3_ = 4.96 · μ_1_ + 0.78 · μ_2_ + 1.48 · μ_3_ + 2.42 · μ_4_ + 55.01 · μ_5_ − 50.63 · μ_6_ − 1.46 · μ_7_ + 5.74 *mf*_4_ = −12.02 · μ_1_ − 13.02 · μ_2_ + 0.26 · μ_3_ − 0.70 · μ_4_ − 5.95 · μ_5_ − 14.74 · μ_6_ − 25.75 · μ_7_ − 25.34 *mf*_5_ = −3 · μ_1_ − 4.78 · μ_2_ + 0.93 · μ_3_ + 3.23 · μ_4_ + 26.66 · μ_5_ − 35.17 · μ_6_ + 0.50 · μ_7_ − 7.78 *mf*_6_ = 5.06 · μ_1_ + 3.27 · μ_2_ + 1.33 · μ_3_ + 1.67 · μ_4_ − 33.29 · μ_5_ − 36.44 · μ_6_ + 8.81 · μ_7_ + 8.33 *mf*_128_ = 3.02 · μ_1_ + 4.11 · μ_2_ + 7.17 · μ_3_ + 0.62 · μ_4_ + 6.18 · μ_5_ + 1.29 · μ_6_ + 6.79 · μ_7_ + 7.13
Score 2 (μ_2_)	0–1	
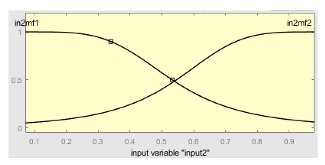		
Predicted Label (μ_3_)	0–1	**Initial configuration**
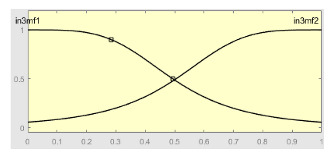		Fuzzy structure: Sugeno-type. Antecedents membership function type: bell-shaped. Consequents membership function type: linear. And method: PROD. Or method: MAX Implication method: MIN. Aggregation method: MAX. Deffuzification method: Weighted average of all rule outputs. Number of fuzzy rules: 128.
Score 1 deviation (μ_4_)	0–0.48	
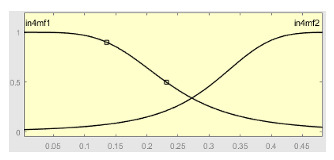		
Score ANFIS 1 (μ_5_)	0–1	**Subset of the 128 fuzzy rules**
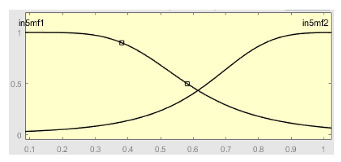		If (input1 is in1mf1) and (input2 is in2mf1) and (input3 is in3mf1) and (input4 is in4mf1) and (input5 is in5mf1) and (input6 is in6mf1) and (input7 is in7mf1) then (output is out1mf1). If (input1 is in1mf1) and (input2 is in2mf1) and (input3 is in3mf1) and (input4 is in4mf1) and (input5 is in5mf1) and (input6 is in6mf1) and (input7 is in7mf2) then (output is out1mf2). If (input1 is in1mf1) and (input2 is in2mf1) and (input3 is in3mf1) and (input4 is in4mf1) and (input5 is in5mf1) and (input6 is in6mf2) and (input7 is in7mf1) then (output is out1mf3). If (input1 is in1mf1) and (input2 is in2mf1) and (input3 is in3mf1) and (input4 is in4mf1) and (input5 is in5mf1) and (input6 is in6mf2) and (input7 is in7mf2) then (output is out1mf4). If (input1 is in1mf1) and (input2 is in2mf1) and (input3 is in3mf1) and (input4 is in4mf1) and (input5 is in5mf2) and (input6 is in6mf1) and (input7 is in7mf1) then (output is out1mf5). If (input1 is in1mf1) and (input2 is in2mf1) and (input3 is in3mf1) and (input4 is in4mf1) and (input5 is in5mf2) and (input6 is in6mf1) and (input7 is in7mf2) then (output is out1mf6).
Score ANFIS 1 deviation (μ_6_)	0–0.73	
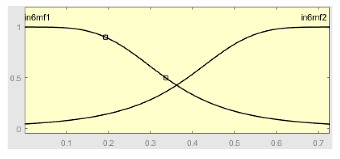		
Correcting factor (μ_7_)	0–1	
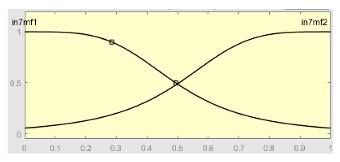		

**Table 9:** Test results for AHI 10.

Models	Metrics	Model Validation	Model Testing	First ANFIS	Second ANFIS
Bagged Trees	AUC	0.87	0.76	0.79	0.84
	MCC	-	0.35	0.43	0.52
Logistic Regression	AUC	0.75	0.79	0.83	0.88
	MCC	-	0.42	0.50	0.57
Coarse Gaussian SVM	AUC	0.75	0.81	0.82	0.86
	MCC	-	0.45	0.48	0.56
Linear SVM	AUC	0.75	0.80	0.82	0.86
	MCC	-	0.45	0.49	0.55
Gaussian Naïve Bayes	AUC	0.73	0.78	0.81	0.86
	MCC	-	0.34	0.49	0.54

**Table 10:** Test results for AHI 15.

Models	Metrics	Model Validation	Model Testing	First ANFIS	Second ANFIS
Bagged Trees	AUC	0.85	0.76	0.78	0.85
	MCC	-	0.40	0.45	0.54
Logistic Regression	AUC	0.75	0.76	0.80	0.86
	MCC	-	0.35	0.47	0.59
Medium Gaussian SVM	AUC	0.76	0.78	0.80	0.88
	MCC	-	0.41	0.47	0.62
Linear SVM	AUC	0.75	0.76	0.78	0.84
	MCC	-	0.37	0.44	0.56
Gaussian Naïve Bayes	AUC	0.72	0.75	0.77	0.84
	MCC	-	0.35	0.42	0.56

**Table 11:** Test results for AHI 20.

Models	Metrics	Model Validation	Model Testing	First ANFIS	Second ANFIS
Bagged Trees	AUC	0.83	0.74	0.77	0.85
	MCC	-	0.30	0.41	0.54
Logistic Regression	AUC	0.73	0.76	0.79	0.85
	MCC	-	0.39	0.47	0.55
Medium Gaussian SVM	AUC	0.74	0.77	0.79	0.83
	MCC	-	0.39	0.46	0.54
Linear SVM	AUC	0.73	0.76	0.77	0.84
	MCC	-	0.40	0.47	0.54
Gaussian Naïve Bayes	AUC	0.71	0.73	0.78	0.84
	MCC	-	0.34	0.46	0.57

**Table 12:** Test results for AHI 25.

Models	Metrics	Model Validation	Model Testing	First ANFIS	Second ANFIS
Bagged Trees	AUC	0.82	0.74	0.76	0.81
	MCC	-	0.33	0.41	0.51
Logistic Regression	AUC	0.73	0.75	0.76	0.82
	MCC	-	0.37	0.41	0.47
Medium Gaussian SVM	AUC	0.74	0.77	0.78	0.83
	MCC	-	0.42	0.44	0.51
Linear SVM	AUC	0.73	0.75	0.75	0.81
	MCC	-	0.35	0.43	0.49
Narrow Neural Network	AUC	0.73	0.72	0.74	0.81
	MCC	-	0.29	0.38	0.47

**Table 13:** Test results for AHI 30.

Models	Metrics	Model Validation	Model Testing	First ANFIS	Second ANFIS
Bagged Trees	AUC	0.84	0.73	0.75	0.81
	MCC	-	0.30	0.39	0.49
Logistic Regression	AUC	0.74	0.77	0.79	0.83
	MCC	-	0.37	0.43	0.50
Coarse Gaussian SVM	AUC	0.74	0.77	0.79	0.86
	MCC	-	0.39	0.44	0.55
Linear SVM	AUC	0.74	0.77	0.78	0.84
	MCC	-	0.35	0.43	0.51
Two-layer NeuralNetwork	AUC	0.74	0.73	0.76	0.84
	MCC	-	0.33	0.39	0.52

**Table 14:** Sensitivity and specificity for each AHI level at the output of the correcting block for the operation set point established from the MCC value.

Threshold Level	Sensitivity	Specificity
AHI 10	0.89	0.67
AHI 15	0.87	0.74
AHI 20	0.87	0.65
AHI 25	0.70	0.81
AHI 30	0.76	0.80

**Table 15:** Data on the patient to be studied.

Data	Values
Gender	Woman
Age	54
Weight	68 kg
Size	152 cm
Neck circumference length	34 cm
Habits	-
Drug treatments	Benzodiazepines and relaxing/hypnotic drugs
Illnesses	Depression

**Table 16:** Diagnostic proposed by the system for 20 cases. Background colors are related with the color of the light indicators associated to the different AHI levels. Red color shows the patient would present the corresponding AHI level. Otherwise, the color is green.

No.	Gender	Age	BMI	NCL	Habits	Drug Treatments	Illnesses	AHI	Results				
1	Woman	70	26.40	34 cm	- Former smoker - 30 packs-per-year	-	- Hypertension	9.70	Statistical inference				
									10	15	20	25	30
									Correcting block				
									10	15	20	25	30
2	Man	71	38.20	41 cm	- Former smoker - 32 packs-per-year	-	- Hypertension - Ischemic heart disease	38	Statistical inference				
									10	15	20	25	30
									Correcting block				
									10	15	20	25	30
3	Man	48	38.93	47 cm	- Former smoker - 36 packs-per-year - Drinking habit: daily, 120g of alcohol	-	- Hypertension	80	Statistical inference				
									10	15	20	25	30
									Correcting block				
									10	15	20	25	30
4	Woman	34	44.29	40 cm	-	-	-	4.50	Statistical inference				
									10	15	20	25	30
									Correcting block				
									10	15	20	25	30
5	Man	41	28.09	36 cm	- Smoker - 10 packs-per-year - Drinking habit: occasionally	- Relaxing/hypnotic drugs- Antidepressants	- Depression	23.40	Statistical inference				
									10	15	20	25	30
									Correcting block				
									10	15	20	25	30
6	Man	39	30.16	41 cm	- Drinking habit: occasionally	-	-	8	Statistical inference				
									10	15	20	25	30
									Correcting block				
									10	15	20	25	30
7	Man	68	30.12	41 cm	-	-	-	26.70	Statistical inference				
									10	15	20	25	30
									Correcting block				
									10	15	20	25	30
8	Woman	66	30.70	34 cm	-	-	- Hypertension - Diabetes	19.70	Statistical inference				
									10	15	20	25	30
									Correcting block				
									10	15	20	25	30
9	Man	62	30.49	46 cm	- Smoker - 104 packs-per-year - Drinking habit: daily, 50g of alcohol	-	-	24.30	Statistical inference				
									10	15	20	25	30
									Correcting block				
									10	15	20	25	30
10	Man	32	32.98	41 cm	- Drinking habit: daily, 50g of alcohol	-	-	26.10	Statistical inference				
									10	15	20	25	30
									Correcting block				
									10	15	20	25	30
11	Man	74	29.36	42 cm	-	-	- Hypertension	37.70	Statistical inference				
									10	15	20	25	30
									Correcting block				
									10	15	20	25	30
12	Woman	48	21.93	33 cm	- Former smoker - 10 packs-per-year	-	-	0.90	Statistical inference				
									10	15	20	25	30
									Correcting block				
									10	15	20	25	30
13	Man	59	52.81	39 cm	- Drinking habit: occasionally	-	-	24.60	Statistical inference				
									10	15	20	25	30
									Correcting block				
									10	15	20	25	30
14	Woman	69	32.07	36 cm	-	-	- Hypertension	27.70	Statistical inference				
									10	15	20	25	30
									Correcting block				
									10	15	20	25	30
15	Man	44	29.76	37 cm	-	-	-	4.90	Statistical inference				
									10	15	20	25	30
									Correcting block				
									10	15	20	25	30
16	Woman	73	24.35	34 cm	-	- Relaxings/hypnotic drugs - Benzodiazepines - Antihistamines	-	17.60	Statistical inference				
									10	15	20	25	30
									Correcting block				
									10	15	20	25	30
17	Man	39	28.73	42 cm	- Smoker - 17 packs-per-year - Drinking habit: occasionally	- Relaxings/hypnotic drugs	-	0	Statistical inference				
									10	15	20	25	30
									Correcting block				
									10	15	20	25	30
18	Man	48	29.03	43 cm	- Smoker - 22.5 packs-per-year - Drinking habit: daily, 30g of alcohol	-	-	61.90	Statistical inference				
									10	15	20	25	30
									Correcting block				
									10	15	20	25	30
19	Woman	52	33.33	37 cm	-	-	-	3.40	Statistical inference				
									10	15	20	25	30
									Correcting block				
									10	15	20	25	30
20	Man	29	41.40	37 cm	- Smoker - 0.5 packs-per-year - Drinking habit: occasionally	-	-	6	Statistical inference				
									10	15	20	25	30
									Correcting block				
									10	15	20	25	30

**Table 17:** Benchmarking.

Methods/Systems	Internal Architecutre	Scalability	Inference	Learning
Corrado Mencar et al. [16], Ramesh et al. [19] and Wen-Chi Huang et al. [20]	The authors’ proposals are based on the use of Machine Learning techniques, highlighting the use of Support Vector Machines in all of them. A probabilistic management of uncertainty is carried out.	The systems are not scalable.	The systems use statistical inference instead of symbolic reasoning.	The proposed system incorporates new knowledge in the process of training the architecture.
Berk Ustun et al. [17]	The authors’ proposal is based on the use of Machine Learning techniques. They highlight the use of Supersparse Linear Integer Models. A probabilistic management of uncertainty is performed.	The system is not scalable.	The system uses statistical inference instead of symbolic reasoning.	The system incorporates new knowledge in the process of training the architecture.
Our proposal	The proposed system manages uncertainty both from a probabilistic and a non-probabilistic point of view.	The proposed system is scalable, since it is possible to modify the calculation engines.	The system uses statistical as well as symbolic inferential approaches, although it does not fully formalize a knowledge base.	The system can incorporate new knowledge as it is being used.

## Data Availability

Not applicable.
